# Efficacy of a single-session online ACT-based mindfulness intervention among undergraduates in lockdown during the COVID-19 pandemic

**DOI:** 10.47626/2237-6089-2020-0172

**Published:** 2022-11-09

**Authors:** Nicholas Tze Ping Pang, Vincent Chung Sheng Tio, Amardeep Singh Bhupendar Singh, Mathias Wen Leh Tseu, Wendy Diana Shoesmith, Muhammad Aklil Abd Rahim, Mohd Amiruddin Mohd Kassim

**Affiliations:** 1 Faculty of Medicine and Health Sciences Universiti Malaysia Sabah Malaysia Faculty of Medicine and Health Sciences, Universiti Malaysia Sabah, Malaysia.

**Keywords:** Mindfulness, online, single-session, anxiety, psychological flexibility, university student

## Abstract

**Introduction:**

COVID-19 has trickle-down psychological effects on multiple strata of society, particularly university students. Apart from the worry of contracting or spreading COVID-19, Malaysian university students were also locked down on their campuses, suffering significant psychological distress. Hence, an online mindfulness intervention was proposed to alleviate psychological distress and improve psychological flexibility and mindfulness.

**Methods:**

This was a quasi-experimental study with university students as participants. Intervention group participants were instructed to complete online questionnaires which covered basic demographics and instruments assessing depression, anxiety, stress, mindfulness, psychological flexibility, and fear of COVID-19 before and after the one-hour intervention. The control group also completed before and after questionnaires and were subsequently crossed over to the intervention group. Repeated measures ANOVA was conducted to assess time*group effects.

**Results:**

118 participants were involved in this study. There were significant differences in anxiety (F_(1,116)_ = 34.361, p < 0.001, partial eta-squared = 0.229) and psychological flexibility between the two groups (F_(1,116)_ = 11.010, p = 0.001, partial eta-squared = 0.087), while there were no differences in depression, stress, mindfulness, or fear of COVID-19.

**Conclusion:**

The results of this study corroborate the efficacy of online single-session mindfulness therapy as a viable short-term psychological intervention under financial and time constraints. Since university students are in the age group with the highest incidence of depressive and anxiety disorders, it is crucial to utilize resources to address as many students as possible to ensure maximum benefit.

## Introduction

The novel SARS-CoV-2 virus, currently known as COVID-19, was first reported in Wuhan, China in December 2019.^1^ Subsequently, it exponentially spread all across the globe, resulting in the World Health Organization (WHO) declaring the disease as a pandemic on 11th March 2020.^2^ Malaysia was not spared, recording the highest number of positive cases in the South East Asia region in March 2020, with the latest figures of 42,872 positive cases and 302 total deaths as of 11th November 2020.^3^

In view of rampant transmission, the Malaysian government enforced strict social and movement restrictions and mandatory quarantine or isolation for persons under investigation and positive cases respectively.^4 , 5^ Although retrospectively swift actions were essential to blunt the progression of spread, nevertheless, such lockdowns have crippled the economy, causing untold social and psychological impairment.^6^ This corroborates with existing literature that psychological illnesses are on the rise during the COVID-19 pandemic.^7 , 8^ Early Chinese data from the initial phase of the outbreak showed that more than half of respondents had suffered moderate to severe psychological impact.^9^ This can be due to direct causes such as stress, fear of contamination, depression, and grief evoked by exposure to the virus as well as indirect consequences of the socioeconomic impact.^10^

Undergraduate students in Malaysia in particular are in unusual circumstances. Due to a sudden explosion in cases related to two less fortuitous coincidences, namely a prison outbreak of Covid-19 and a concurrent general election in the same state,^11^ students in Sabah were uniquely locked down on campus and all teaching and learning was converted to online mode, whereas their counterparts in all other universities in Malaysia were allowed to continue face to face teaching and learning as usual. This was especially compounded in the sample population investigated in this study, because they were all medical students. Since the medical degree program is a highly vocational course of study, it is by nature highly dependent on bedside teaching and face to face work.^12 - 14^ Hence, for medical students who are locked down physically and educationally with no conceivable end in sight, there may be a strong sense of loss of purpose,^15^ perceived loss of ability and skillset,^16^ and potential recriminations or self-blame,^17^ especially for those who have friends at other medical universities in other parts of the country that have not been locked down. As such, there is a high possibility of psychopathologies developing.^18^ Such psychological issues can present a major obstacle to academic performance, which can affect students’ concentration, motivation, and social interactions, which all play vital roles in enabling students to succeed in their higher education.^19 , 20^ Recent intra-pandemic data indicate that Covid-19 has negative impacts on higher education such as fear of infection of self and loved ones, difficulty concentrating, sleep disruptions, reduced social interactions, and worse academic results.^21 , 22^ It is therefore instrumental to prevent such deteriorations from perpetuating further and interventions with known efficacy can be employed to serve as crucial public health primary prevention interventions to promote psychological wellness in quarantined and anxious university students.^23^ Mindfulness-based interventions constitute one such intervention with known efficacy and can increase mindfulness and psychological flexibility.^24 , 25^

Mindfulness can be defined as attending to one’s present-moment thoughts, feelings, and sensations, with an open and curious mind, and without attempting to change the experience.^26^ Mindfulness techniques are currently incorporated into many therapies in mental health care, namely mindfulness-based stress reduction (MBSR), mindfulness-based cognitive therapy (MBCT), dialectical behavior therapy (DBT), and acceptance and commitment therapy (ACT).^27^ Psychological flexibility is a similar construct involving the ability to fully experience the present moment, including observing one’s thoughts and feelings, without struggling to control or change them, and the ability to either persist or change behavior in the given context in a manner that is consistent with one’s values and goals.^28^ Hence, mindfulness techniques can be employed to assist with all three aspects of psychological flexibility. They enable the aforementioned observation of one’s thoughts impartially and non-judgmentally, which then allows full and rich experiences of the present moment. An individual is thus able to persist with value-congruent behavior, because mindfulness techniques facilitate acceptance of the inevitable accompanying pain.^29 , 30^ Literature shows that mindfulness and psychological flexibility are related in terms of psychological distress,^31^ and psychological distress is significantly reduced in individuals with higher psychological flexibility.^28^

Due to the distancing requirements during this pandemic, there has been renewed interest in development and assessment of the use of digital and online tools as viable alternatives for care delivery.^32 - 34^ Online mindfulness meditation (MM) training has demonstrated equal benefits to face-to-face MBSR training for reduction of depression, stress, and anxiety.^35 , 36^ Culturally closer to home, evidence from Singapore reports similar effectiveness for mindfulness training conducted through videoconferencing and traditional in-person modalities.^37^ In the specific context of university students, there were significant reductions in depression and anxiety, but not stress, after web-based mindfulness performed over 8 weeks.^38^ Similar evidence showing that online interventions reduced psychological stress, enhancing mental health, sleep disturbance, life satisfaction, and energy level was demonstrated in college students and working adults.^39 , 40^ However, in the recent pandemic, there has been no evidence from Malaysian settings that corroborates the aforementioned findings. There are however descriptive studies of ultra-brief psychological intervention modules that adapt mindfulness techniques, allowing them to be delivered in shorter-format single interventions outside of formalized psychotherapeutic protocols.^41^ From a public health perspective, with finite psychological resources, running a single-session protocol with proven efficacy delivers far greater benefits from a utilitarian perspective than only delivering multi-session tertiary interventions for individuals with known psychological morbidity. The objectives of this project were to assess the efficacy of a single-session mindfulness intervention adopted from ACT and measure changes in levels of general and Covid-19-related psychopathology. The hypotheses were that there would be lower levels of depression, anxiety, and stress, and higher levels of psychological flexibility and higher levels of state mindfulness after a single session mindfulness-based intervention, compared to a matched control group.

## Methodology

### Ethics

Ethical approval was obtained from the Universiti Malaysia Sabah Medical Research Ethics Committee prior to commencement of this project. All participants provided informed consent.

### Study design and setting

This was a quasi-experimental study conducted with university students during the Malaysian Conditional Movement Control Order (CMCO) period, when social gatherings were not permitted. During the CMCO period, participants attended a one-hour single-session online mindfulness intervention. Questionnaires were answered before and after the online session. Since this was a psychological intervention, it was impossible to blind participants, therefore both intervention and control groups received the same intervention at different times. The intervention group attended the session prior to the posttest, while the control group attended it afterwards.

### Participants and sample size

By applying the nonequivalent groups design approach, and to ensure both groups were as similar as possible, equal numbers of participants from batches of undergraduate medical students were divided into intervention and control groups based on each year’s student roll. The sample size calculation was based on a formula described by Lehr,^42^ using a significance criterion of 0.05, statistical power of 0.8, and effect size of 0.63.^43^ The required sample size was 41 in each group, hence 82 participants cumulatively. The inclusion criteria were university students over the age of 18 who were locked down on campus, were willing to participate in the study, and were able to read and converse fluently in Malay. The exclusion criteria were non-consent and acute medical or psychiatric illness, which was obtained from the electronic medical records of the university hospital and corroborated with self-reported symptoms as a second-level safety net.

### Questionnaires

#### Fear of Covid-19 Scale (FCV-19S)

The Fear of Covid-19 Scale^44^ consists of seven items (e.g. “It makes me uncomfortable to think about coronavirus-19”). It is scored on a five-item Likert response scale ranging from 1 (strongly disagree) to 5 (strongly agree), with possible scores ranging from 7 to 35. Higher scores indicate more severe fears of COVID-19.^44 , 45^ In this study, a validated Malay version^46^ was administered that has very good internal consistency, with Cronbach alpha of 0.893 and McDonald’s omega of 0.894.^46^

#### Depression anxiety and stress scales 21-item (DASS-21)

The DASS-21^47^ measures the severity of depression, anxiety, and stress. It consists of 21 items that measure three different domains: depression (e.g. “I felt downhearted and blue”), anxiety (e.g. “I was worried about situations in which I might panic and make a fool of myself”), and stress (e.g. “I found it hard to calm down after something upset me”). Each item is scored on a four-point Likert scale ranging from 0 (did not apply to me at all over the last week) to 3 (applied to me very much or most of the time over the past week). Higher scores in each domain correlate with greater severity of emotional distress. In this study, the Malay version of the DASS-21^48^ was administered, which has achieved acceptable Cronbach’s alpha values of 0.84, 0.74 and 0.79, respectively, for the depression, anxiety and stress scales, as well as a Cronbach’s alpha of 0.904 for the overall score. In addition, it had good factor loading values for most items (0.39 to 0.73).^48^

#### Acceptance and Action Questionnaire (AAQ-II)

The AAQ-II^49^ is a widely-used measure of experiential avoidance and psychological inflexibility. It was developed and revised from the original AAQ.^50^ It is a unidimensional scale with 7 items rated on a 7-point Likert scale ranging from 1 (Never true) to 7 (Always true). Possible scores range from 7 to 49. Higher scores on the AAQ-II indicate higher levels of psychological inflexibility. The Malay version of AAQ-II used in this study has a Cronbach alpha of 0.910, excellent parallel reliability, and adequate concurrent validity.^51^ We elected to use the unifactorial AAQ-II instead of the longer equivalents because the extant literature suggests that it is psychometrically superior and thus more reliable and valid.^49 , 51^

#### Mindfulness, Attention, and Awareness Scale (MAAS)

The Mindful Attention Awareness Scale (MAAS) is used to assess awareness and attention in everyday life. It is a 15-item scale that measures the frequency of mindful states in day-to-day life using both general and situation-specific statements. A range of scores between 1 to 6 are allocated for each item. Totals range from 15 to 90, with higher scores indicating greater mindfulness.^26^ In this study, the Malay version of the MAAS (MMAAS) was administered. The MMAAS has good internal consistency (Cronbach’s α = 0.851) and satisfactory psychometric properties.^52^

## Procedure

All interactions were online because social contact was not permitted. After the respondents had been divided into groups, they were presented with study information and gave consent. The intervention group was required to complete 2 sets of questionnaires, one before the intervention and one after the intervention. These were the Fear of Covid-19 Scale (FCV-19S), the Depression Anxiety and Stress Scales 21-item (DASS-21), the Acceptance and Action Questionnaire (AAQ-II), and the Mindfulness, Attention, and Awareness Scale (MAAS).

The pre-intervention forms were completed an hour before the intervention. After completing the pre-intervention forms, members of the intervention group participated in an hour-long online mindfulness session conducted via the Google Meet videoconferencing platform (https://meet.google.com/). The session was led by a moderator (Clinical Psychiatrist) and was based on the mindfulness components of the ultra-brief psychological interventions manual, which adopts ACT techniques.^41^ All participants were involved in a group session online, but each was able to receive individualized feedback as required. The size of each group was 30 individuals.

During the first five minutes of the session, the moderator began by explaining the core principles behind mindfulness and tying it in to the three processes underlying psychological flexibility. Next, over the subsequent 45 minutes, the moderator demonstrated 3 different mindfulness techniques, allocating roughly 15 minutes to each technique: (1) the breathing technique, (2) the tactile sensation technique, and (3) the focusing on a specific musical instrument technique, all in relation to psychological flexibility. All three techniques were then practiced in smaller breakout rooms of 5-6 people, with various participants demonstrating to the moderator how they would perform it, and the moderator giving them feedback for improvement and periodically relating the practical activities back to psychological flexibility. The final 10 mins of the session were used for a Question and Answer (QnA) session during which the participants could raise any doubts and discuss their concerns. After the intervention, the respondents were required to fill in the post-intervention questionnaire at a time corresponding to 24 hours after the online session. The control group filled in the pre-intervention and post-intervention questionnaires at the same times as the intervention group and then subsequently crossed over to participate in the same intervention.

## Data analysis

Data were analyzed according to the intention-to-treat principle. All participants were analyzed according to the condition (Intervention or Control) they were initially allocated to. IBM SPSS version 26.0 was employed for all statistical analysis. Descriptive statistics were used, with skewness and kurtosis calculated to assess whether normality criteria were met. Subsequently, repeated measures ANOVA was performed to assess whether there were differences between control and intervention groups. F values were reported with significance at p < 0.05 and partial eta-squared was also reported to indicate effect size. Estimated marginal means for each measured parameter were plotted on a graph for two time points – pre-intervention (T1) and post-intervention (T2).

## Results

A total of 118 participants volunteered for the mindfulness intervention. They were divided into intervention and control groups, containing 61 and 57 participants respectively. The demographics of the students are as described in Table 1 . Table 2 illustrated that all continuous data for depression, anxiety, stress, FCV-19S, MAAS, and AAQ-II scores were normally distributed with skewness and kurtosis within appropriate ranges (+/-2 and +/-10 respectively) as per normality requirements.^53^

**Table 1:** Demographics of the participants

	Frequency	Percent
Gender		
Female	74	62.700
Male	44	37.300
Total	118	100.000
Age	104	35.600
19-25	13	0.800
31-35	1	0.800
42	118	100.000
Total		22.310
Mean		
Standard deviation		4.523

**Table 2:** Descriptive statistics for the results (intervention and control)

	FCV-19S pre-test score	FCV-19S post-test score	Depression pre-test score	Depression post-test score	Anxiety pre-test score	Anxiety post-test score	Stress pre-test score	Stress post-test score	AAQ-II pre-test score	AAQ-II post-test score	MAAS pre-test score	MAAS post-test score
Intervention (N = 61)												
Skewness	1.148	1.806	0.812	2.665	1.666	1.606	1.026	1.219	0.796	1.803	-0.694	-1.028
Kurtosis	1.852	3.964	-0.570	9.676	2.569	2.593	0.662	0.645	-0.765	3.294	-0.764	-0.333
Control (N = 57)												
Skewness	-0.100	0.318	0.960	1.258	0.651	0.875	0.695	0.641	0.617	0.973	-0.401	-0.428
Kurtosis	-0.849	-0.626	0.415	1.018	-0.562	0.105	-0.236	-0.045	-0.003	0.907	-0.880	-0.880

AAQ-II = Acceptance and Action Questionnaire; FCV-19S = Fear of Covid-19 Scale; MAAS = Mindfulness, Attention, and Awareness Scale.

As shown in Table 3 , normality checks were carried out on the residuals for FCV-19 scores and depression, stress, and mindfulness scores, which were all approximately normally distributed. A repeated measures ANOVA showed that there was no significant difference between control and intervention groups for any of these 4 variables.

**Table 3:** Tests for within-subjects effects

Measure	Type III sum of squares	Df	Mean square	F	Sig.	Partial eta squared
Depression	1.714	1	1.714	0.637	0.426	0.005
Anxiety	223.716	1	223.716	34.361	0.000	0.299
Stress	8.970	1	8.970	2.859	0.094	0.024
Fear of Covid-19	0.741	1	0.741	0.117	0.733	0.001
Psychological flexibility	143.781	1	143.781	11.010	0.001	0.087
Mindfulness	132.826	1	132.826	1.391	0.241	0.012

Source: test group.

* Sphericity assumed in all measures.

**Figure 1:**
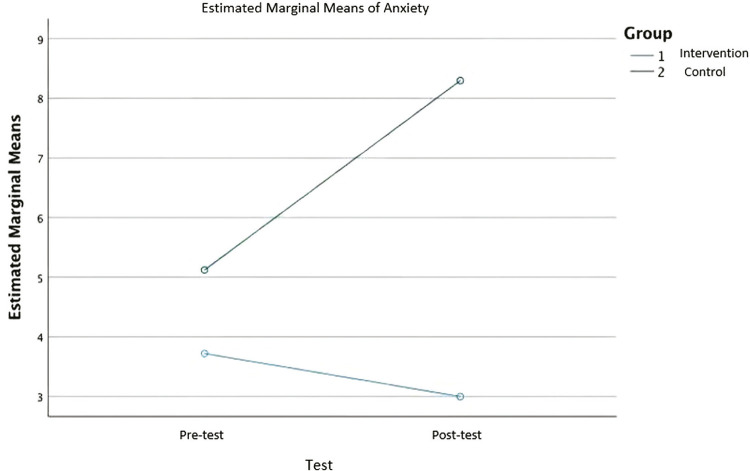
Estimated marginal means for anxiety

Normality checks were also carried out on the residuals for anxiety scores, which were approximately normally distributed. A repeated measures ANOVA showed that the anxiety scores differed significantly between control and intervention groups, F_(1,116)_ = 34.361, p < 0.001, partial eta-squared = 0.299 ( Figure 1 ).

**Figure 2:**
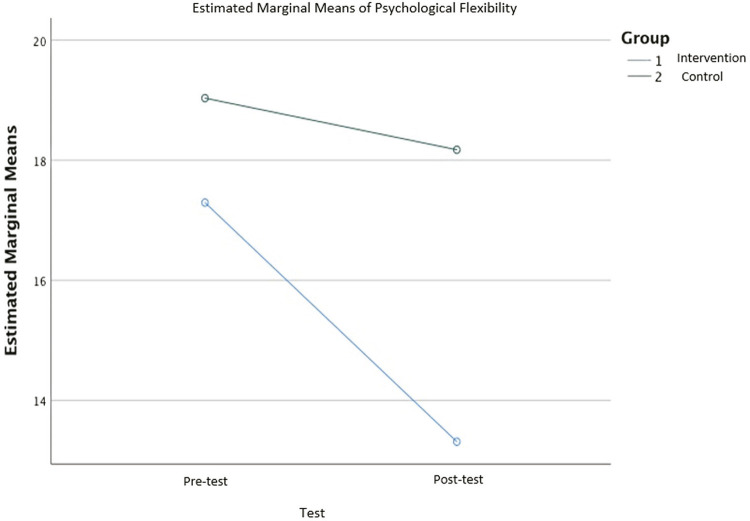
Estimated marginal means for psychological flexibility.

Normality checks were carried out on the residuals for psychological flexibility scores, which were approximately normally distributed. A repeated measures ANOVA showed that psychological flexibility differed significantly between control and intervention groups, F_(1,116)_ = 11.010, p = 0.001, partial eta-squared = 0.087 ( Figure 2 ).

## Discussion

This study demonstrates key findings, namely that a single-session online mindfulness intervention adopting ACT principles demonstrated efficacy in terms of reducing anxiety and improving psychological flexibility. This is also reflected in the literature, where other online single-session interventions have been equally efficacious.^54 - 56^ This again underscores the importance of psychological flexibility as a construct that can be worked on in a pandemic situation. In view of the never-ending nature of the successive waves of Covid-19 and the uncertainty affecting almost all aspects of life, be it national quarantine and lockdown regulations, standard operating procedures, ability to travel, and/or the possibility of pursuing education and relationships, it is crucial that this intervention can boost psychological flexibility significantly, in order to enable people to achieve less rigid, more experientially open thought processes regarding the unpredictable and uncontrollable future.^57^

Interestingly, there were no effects for depression, stress, fear of Covid-19, or mindfulness scores. This suggests that anxiety may be the key construct that is significantly higher in the population,^58 , 59^ and therefore more amenable to reduction. Also, it may suggest that a longer number of sessions might be needed to show significant reductions in the above constructs. However, we need to balance cost effectiveness in terms of providing one efficacious intervention to more people versus many more efficacious interventions, but to fewer people. Moreover, the results of this study echo findings from other literature that suggests that single session interventions are of equal efficacy to longer multi-session ones.^60 , 61^

This study certainly has certain limitations. Firstly, all of the participants are medical students, who might have higher awareness and knowledge regarding updates about COVID-19, compared to other students on different courses or to the general public.^62 - 64^ This could therefore represent a selection bias. Secondly, the sample size of 118, including both the intervention and the control group, was relatively small, partly due to internet access limitations and time constraints and also because three batches of medical students had already undergone mindfulness intervention training pre-research, thus raising the possibility that their inclusion would unduly distort the final results. As it stood, only 183 students had not undergone mindfulness training before. One more limitation includes the lack of an active control group. The significant effects on reduction of anxiety and increasing psychological flexibility may have simply been caused by the fact that there was interaction with a caring and supportive professional, rather than being related to any specific effect of the mindfulness technique used.

On the other hand, the absence of effects on the other scales may also simply represent the need for sustained practice to modify the aspects measured. This can be tested with one or more follow-up assessments. Lastly, since the participants were locked down on campus and staying in proximity to each other in the hostel, it was difficult to blind participants and there was a possibility of the intervention group participants sharing the techniques taught during the mindfulness intervention with the control group, hence affecting the results.

In conclusion, the results of this single-session intervention study are crucial because there is a need for evidence-based short interventions that can be provided to the largest number of people to yield the greatest good, rather than for multiple-session interventions which no doubt have unquestionably higher efficacy due to the additive effect of multiple sessions, but are exhaustive to run and have high attrition rates, and may result in fewer individuals getting the benefits of mindfulness training and thus have less public health impact.^65^ ACT techniques have been successfully delivered in single-session modalities with good evidence for multiple indications^66 - 70^ and it is especially crucial we offer them to undergraduate populations, because they are in the age group with the highest incidence of depressive and anxiety disorders.^71^ Therefore, it is our greatest hope that this single session intervention will be adopted in other universities as this intervention has shown limited efficacy, albeit in a single center, and larger sample sizes would be ideal to explore the efficacy of this intervention further.
